# Retrospective validation of SATURN, a public domain self-administered cognitive screening test

**DOI:** 10.3389/fpsyg.2026.1801901

**Published:** 2026-05-18

**Authors:** Katrina Ang Pasao, Maria Noelle Florendo, David Bissig

**Affiliations:** Department of Neurology, University of California, Davis, Sacramento, CA, United States

**Keywords:** cognitive screening, computerized testing, dementia, mild cognitive impairment, self-administered

## Abstract

**Introduction:**

Mild cognitive impairment and dementia are often underdiagnosed. Self-Administered Tasks Uncovering Risk of Neurodegeneration (SATURN) is a public domain computerized cognitive screening test developed to address that need. We describe its performance in regular clinical use.

**Methods:**

We retrospectively compared scores of SATURN with those from paper-and-pencil screening instruments—the Montreal Cognitive Assessment (MoCA), the Mini-Mental State Examination (MMSE), the Saint Louis University Mental Status (SLUMS), and the Functional Activities Questionnaire (FAQ)—and calculated receiver operating characteristic curves relating each to clinician diagnosis.

**Results:**

SATURN was strongly correlated with MoCA (*r* = 0.82), MMSE (*r* = 0.76), SLUMS (*r* = 0.81) and inversely correlated with FAQ (*r* = −0.55). SATURN demonstrated the best area under the curve (0.95) for distinguishing cognitively normal from cognitively impaired patients and ranked higher at distinguishing individuals with dementia and individuals without dementia (SLUMS > SATURN > MoCA = MMSE > FAQ).

**Discussion:**

SATURN compared favorably compared with established cognitive screening instruments in regular clinical practice.

## 1 Introduction

Early detection of cognitive impairment is critical for appropriate care and timely treatment; however, it remains underdiagnosed (Mattke et al., 2023b). Neuropsychological research has demonstrated that cognitive impairment, even at its earliest stages, can significantly affect real-world functional abilities such as independent activities of daily living and financial decision-making. This emphasizes the need for scalable cognitive screening tools that accurately assess multiple cognitive domains, including memory, executive functioning, and judgment, and identify individuals at high risk of functional decline (Giannouli, 2023b). Paper-and-pencil cognitive screening tools, such as the Mini-Mental State Examination (MMSE), the Montreal Cognitive Assessment (MoCA), and the Saint Louis University Mental Status (SLUMS) exam, were developed to aid in the identification of cognitive impairment, particularly during the earliest stages of the disease (Nasreddine et al., 2005; Tariq et al., 2006).

Unfortunately, these cognitive tests remain underutilized: In a recent study, only 14% of patients diagnosed with dementia in primary care had been administered one of the three aforementioned tests (Nguyen et al., 2022). The clinician’s time typically spent testing is a barrier to greater use, warranting interest in screening by non-clinicians (Mattke et al., 2023a; Hilgeman et al., 2019) or self-administered testing (Scharre et al., 2010). Scoring errors are common with such approaches (Hilgeman et al., 2019; Scharre et al., 2024), arguing for computerized, automatically scored testing. Several instruments were developed in response to this need; however, the majority of them lack data on the feasibility of use in their intended clinical setting (Tsoy et al., 2021) and introduce other barriers, such as cost or limited availability in other languages.

Self-Administered Tasks Uncovering the Risk of Neurodegeneration (SATURN) is a self-administered, automatically scored screening test developed to remove barriers to cognitive screening. In its initial prospective validation study, SATURN and MoCA scores were strongly correlated (*r* = 0.90), and both tests predicted illness severity on the Clinical Dementia Rating scale (Bissig et al., 2020b). SATURN is freely available (Bissig et al., 2020a) in the public domain, allowing it to be shared, used, and adapted by all (Bissig et al., 2020b). Collaborators have demonstrated the fully remote use of SATURN (Tagliabue et al., 2023) and developed a German smartphone version, digiDEM-SCREEN (Zeiler et al., 2025a), allowing patients to be screened in their own homes. In the unsupervised collection of Italian-language norms, the limited assistance of the test-taker did not affect any outcomes (Giaquinto et al., 2024). Draft versions in other languages (Bissig et al., 2020a; Truong et al., 2024) further expand SATURN’s utility. Despite these efforts, to date, there is no description of SATURN’s long-term deployment in a clinical setting. We, therefore, initiated this retrospective study of SATURN’s ongoing use in a single clinic, testing the hypotheses that SATURN scores are well correlated with and have similar diagnostic value to the aforementioned legacy tests (MMSE, MoCA, and SLUMS).

## 2 Methods

### 2.1 Patient selection and data extraction

**Figure 1:**
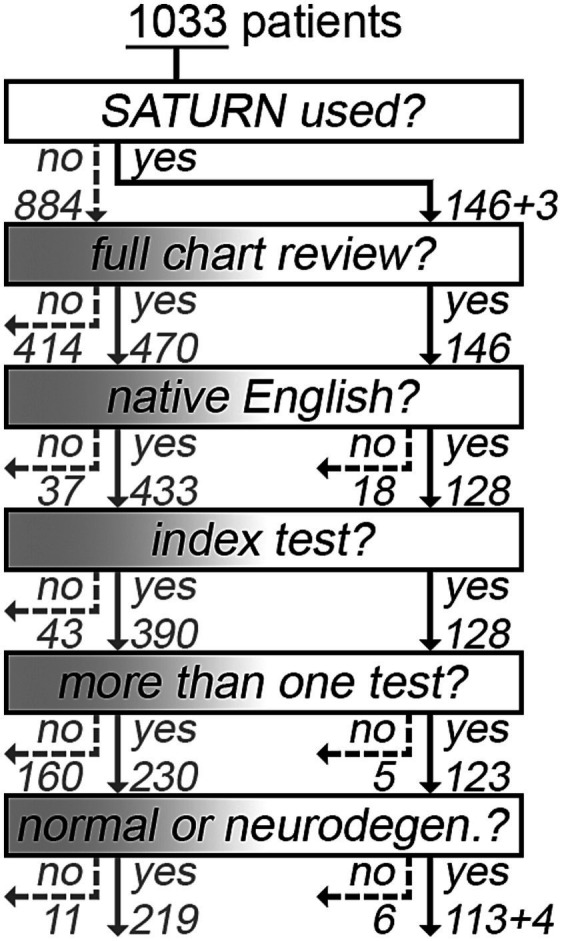
Flow diagram describing the chart selection for the primary analyses. According to our analysis plan, we manually reviewed all 146 patients tested using SATURN during a clinic visit from August 2020 to July 2022 (right side of figure). The “+3” at the top denotes three cases where SATURN appeared in the patient chart, but it was used for unrelated research. Exclusions occurred due to not being a native English speaker (*n* = 18) or for having only one of the four cognitive tests being compared (*n* = 5 were not tested using the MMSE, MoCA, or SLUMS within 365 days of the index event). Among SATURN users, the “+4” denotes patients who were “unscorable” as defined in the original validation study (Bissig et al., 2020b), which signals the presence of dementia, but without enough numerical information to contribute to regression analyses of SATURN vs. other test scores. Based on the analysis plan, we also conducted a full manual chart review of 470 patients who were never tested using SATURN (left side of figure), who were similar to the remaining 414 patients (Supplementary Table S1). After excluding non-native English speakers (*n* = 37), 43 did not have an “index test”: For 12 patients, the Self-Administered Gerocognitive Exam (SAGE) (Scharre et al., 2010) or the Short Blessed Test (Katzman et al., 1983) was used instead of the MMSE, MoCA, SLUMS, or SATURN. Another four had no cognitive complaints (erroneous referrals). For another 17 patients, dementia was advanced enough that no test was attempted. Testing was abbreviated for the remaining 10 patients (e.g., due to sensory loss). Of the remaining, only 230 patients had one other test score (e.g., a MoCA or SLUMS, if the index test was an MMSE). Finally (figure bottom), we excluded patients who were neither cognitively normal nor had a neurodegenerative process. They typically either had a primary psychiatric disorder accounting for cognitive complaints (10/17) or were experiencing sequelae of traumatic brain injury (3/17).

We retrospectively analyzed the medical records of patients evaluated for a primary neurodegenerative disorder from August 2020 to July 2022 in our dementia practice. For that time period, we manually reviewed all 146 patients who were tested with SATURN during their clinic visit, which was noted as the “index event.” Re-testing with SATURN within the prespecified time range was uncommon; therefore, only its first use was considered. For patients who were never tested with SATURN, the “index event” was the first visit at which a score was obtained on the MMSE, MoCA, or SLUMS. We excluded patients who did not undergo any of the other “index tests” at our clinic (Figure 1). In an interim analysis of the first 92 patients analyzed for this study (Florendo and Bissig, 2023), the correlations between SATURN and each of the other tests were strong enough (*r* ≥ 0.8) that other considerations guided the total sample size. We reviewed the correlations between the MMSE, MoCA, and SLUMS because this contextualizes the correlations between SATURN and each of those tests. The weakest correlation in that an interim analysis (SLUMS vs. MMSE; *n* = 9, *r* = 0.48) led us to predict that (i) after exclusions, 31 patients with that combination of tests would be needed to detect a correlation (*α* = 0.05) with a power of 0.8; however, (ii) only approximately 10% of the total would have that combination of tests (a SLUMS and an MMSE score). We therefore set a target of approximately 310 patients to review after exclusions, and when presenting that interim analysis, we reported our plan to conduct a full chart review of approximately 600 charts. We proceeded as planned (full chart review of 146 tested using SATURN and 470 of the others, total, *n* = 616; Figure 1) and slightly exceeded the total number needed after exclusions (*n* = 336; Figure 1, bottom).

Patient age and diagnosis were logged for the index event. If a patient was documented as being “on the border” between two diagnoses (e.g., mild cognitive impairment and mild dementia), we logged the less severe diagnosis. We excluded patients with cognitive impairment due to a non-neurodegenerative process (Figure 1). Raw data are provided alongside detailed terms and definitions in a data repository (Pasao et al., 2025). Sex, ethnicity, education, and etiologic diagnoses (Table 1) were logged as best understood at the time of data extraction. Etiological classifications were based on the dementia subspecialist’s (author D.B.’s) best clinical diagnosis using standard criteria and available clinical information. Aside from the above data elements, we censored information created after 31 July 2022. For the period of ±365 days from the index event, we also logged the following when available: written diagnosis from any formal neuropsychological assessment, any use of the Functional Activities Questionnaire (FAQ) (Pfeffer et al., 1982)—which is often handed to a person accompanying the patient during clinic check-in—and up to two scores on each legacy screener (MMSE, MoCA, or SLUMS) by any provider. Having up to two scores on each legacy screener allows test–retest comparisons for each test, capturing variability over time due to medical events or disease progression. Unless otherwise specified, statistical comparisons used the test closest in time to the index event.

**Table 1:** Demographics of patients used in primary analyses.

		Tested using SATURN?		*p-*value	Effect size
		No	Yes		
Patients	#	219	117		
Age	yr	74 (10)	76 (9)	0.019	0.27
Women	%	58	40	0.002	0.17
Ethnicity documented	%	96	98	0.4	0.05
African-American, Asian, White, Other^*^	%	3,5,86,5	7,4,86,3	0.3	0.10
Ethnicity documented	%	93	97	0.1	0.08
Ethnicity: Hispanic/Latino	%	5	4	0.6	0.03
Education documented	%	96	97	1.0	0.00
Education	yr	15 (3)	15 (3)	0.7	0.04
<12 years	%	3	3		
≥16 years	%	48	51		
≥18 years	%	25	20		
Cognitively normal, MCI, and dementia	%	16,36,48	20,41,39	0.3	0.08
Etiology documented	%	66	62	0.5	0.04
Alzheimer’s disease^†^	%	55 [66]	43 [60]	^‡^ > 0.1	^‡^ < 0.11
*α*-synucleinopathy^†^	%	6 [9]	14 [19]		
Vascular^†^	%	15 [24]	8 [19]		
Tauopathy or TDP-43 proteinopathy	%	4	10		
Mixed	%	13	21		
Other	%	7	4		

Other than counts (#), values are percentages and means (standard deviations); effect size is Cohen’s *w* or Cohen’s *d*, respectively.

* Combines chart entries of “Other” and entries with prevalence <2%.

† Percentage in brackets counts the presence of that etiology in mixed cases.

‡ Chi-squared test for Alzheimer’s disease vs. non-Alzheimer’s disease (*p*-value varies with the inclusion of mixed cases).

The decision to use SATURN, MMSE, MoCA, or SLUMS was typically made the day before the clinic visit. We were more likely to use SATURN when another test result was already on file, which explains the small proportion of SATURN cases excluded from having more than one test (Figure 1). On the other hand, any report of interval cognitive decline favored reusing the same legacy test to characterize the clinical trend. In our clinic, the MMSE is favored when dementia manifests.

This is a retrospective analysis of real-world clinical practice, and the scope of data extracted from each chart is broad—including, *when available*, scores from SATURN and FAQ; two scores for the MMSE, MoCA, and SLUMS; and diagnosis from formal neuropsychological testing. We therefore expect each patient to have some missing data elements and for the data to be missing not-at-random (MNAR). For instance, clinicians trending MMSE and SLUMS scores (four patients who underwent full chart review had two scores for both tests) would be unlikely to trend the same patients’ MoCA scores during the ±365-day review period. We indeed found no such cases. Consistent with best practices for frequent MNAR data, we avoided multiple imputation, used only observed data, and provided clear details about the extent and nature of missing data (Jakobsen et al., 2017). To this end, (i) we compared available data on those who received and did not receive a full chart review (Supplementary Table S1) and incidentally showed that a full manual chart review clearly reduced missingness; (ii) we comprehensively collected cognitive testing data in our sample by logging SATURN, MMSE, MoCA, and SLUMS scores. Cases lacking one test are still well characterized by the other tests. (iii) We detailed the exclusions from the planned analyses (Figure 1) and reported sample sizes for each analysis. Most importantly, (iv) we provided our full dataset via a Dryad repository (Pasao et al., 2025).

### 2.2 Statistical analysis

We used R statistical software (version 4.1.1) for all analyses. In our primary analyses, we tested the relationship between scorable (Bissig et al., 2020b) SATURN results and the legacy screening tools (MMSE, MoCA, SLUMS, and FAQ) with a linear regression analysis and used a one-way analysis of variance (ANOVA) with Tukey’s method to compare each tool’s scores to neurologist diagnosis: normal vs. mild cognitive impairment (MCI) vs. dementia.

Demographic data were compared using *t*-tests for continuous variables and chi-squared tests for others. For comparisons of SATURN to another score, we tested for non-linearity by adding a quadratic term and dropping it from subsequent analyses if the *p*-value was> 0.05. In those regression models, we also examined the effects of including age, sex, level of education, or ethnicity as a covariate. In the Supplementary material, analyses are contextualized by inter-test and test–retest correlations for the MMSE, MoCA, and SLUMS.

We used receiver operating characteristic (ROC) analyses to describe each tool’s ability to distinguish those without impairment (normal) from those with impairment (MCI or dementia) and to distinguish those without dementia (normal or MCI) from those with dementia. In the Supplement, we repeated those analyses using—where available—the diagnosis from a formal neuropsychological assessment. Assuming that precision-recall (PR) curves are sometimes used alongside ROC curves to assess test performance (Richardson et al., 2024), these are also calculated in the Supplement.

One may want to use SATURN to predict a patient’s MoCA, MMSE, or SLUMS score. To account for greater uncertainty at lower scores, which occurred less often in our sample, we re-ran regression analyses and calculated 50% prediction intervals after a power transformation. Predicted values were rounded to the nearest integer. To judge the quality of this approach, we applied it to preexisting repository data relating SATURN to the MoCA (Bissig et al., 2020b), testing its ability to predict the present data, and vice versa.

### 2.3 Statement of ethics

The study was exempted by the University of California - Davis Institutional Review Board (IRBNet ID # 1946437-2). Being a retrospective chart review, it was exempt from requiring informed consent.

## 3 Results

### 3.1 Sample demographics/characteristics

A total of 1,033 patients were included in the study, with exclusion criteria shown in Figure 1. Of those not tested with SATURN, we found that the 470 patients receiving a full manual chart review were representative of the 414 who were not reviewed in that depth (Supplementary Table S1).

For the primary analyses—using 117 tested with SATURN and 219 not tested with SATURN—those tested with SATURN were slightly older and more likely to be men (Table 1), but the groups were otherwise statistically similar. Post-hoc, we found that women (65%) were more likely than men (52%) to have been tested (MMSE, MoCA, or SLUMS) by another provider before the index event (*Χ*^2^ (1, *N* = 336) = 5.48; *p* = 0.019). This, in turn, would influence the choice of test during our clinic encounter.

### 3.2 Relationship to neurologist diagnosis

**Figure 2:**
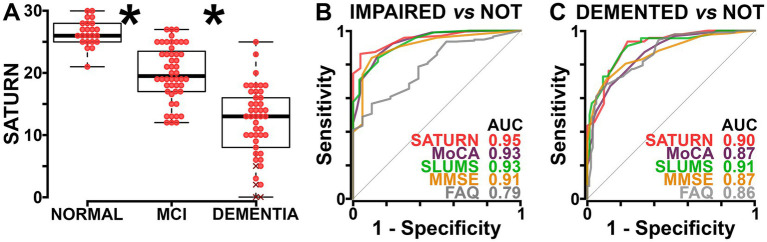
Self-Administered Tasks Uncovering the Risk of Neurodegeneration (SATURN) clearly identifies patients with mild cognitive impairment (MCI) and dementia. **(A)** A beeswarm plot overlaid on a boxplot showing the significant (one-way ANOVA, *F*_[2,_ 1_14]_ = 78.12, *p* < 0.001, *η*^2^ = 0.58) association between SATURN score and neurologist diagnosis. The Xs denote four “unscorable” patients, as previously defined (Bissig et al., 2020b). While not used in regression analyses, an “unscorable” SATURN test supports the diagnosis of dementia. Using Tukey’s honestly significant difference test, we found robust differences between SATURN scores of normal vs. MCI groups (*p < 0.001; Cohen’s *d* = 1.69), MCI vs. dementia groups (*p < 0.001; *d* = 1.55), and normal vs. dementia groups (*p* < 0.001; Cohen’s *d* = 2.95). Similar plots for the other tests—the Montreal Cognitive Assessment (MoCA), Mini-Mental State Exam (MMSE), Saint Louis University Mental Status (SLUMS) exam, and Functional Activities Questionnaire (FAQ)—are provided in the supplement (Supplementary Figure S1). **(B)** Receiver operating characteristic (ROC) curves show each test’s relationship to a neurologist’s diagnosis of having no impairment (normal) vs. having cognitive impairment (MCI or dementia). Numerically, SATURN has a higher area under the curve (AUC) than the other tests. Optimal cutoff scores according to the ROC analyses and precision-recall analyses are provided in the supplement (Supplementary Table S2). **(C)** ROC curves, such as those in **(B)**, but contrasting those without dementia (normal or MCI) to those with dementia.

SATURN scores were robustly associated with clinical diagnosis (*p* < 0.001; Figure 2). In ROC analyses, SATURN had the numerically highest ROC area under the curve (AUC) when comparing normal and impaired patients. It also ranked well in distinguishing individuals with dementia from those without dementia (Figure 2; Supplementary Table S2).

Those tested with SATURN were not always tested with a specific legacy test (and vice versa). For each legacy test, we therefore repeated ROC analyses on the subset of patients tested with both SATURN and that legacy test. ROC AUCs for SATURN often trended higher than those for each legacy test; however, the only statistical difference was SATURN’s superiority over the FAQ at distinguishing cognitively normal patients from those with impairment (*p* = 0.0045; Supplementary Table S4).

### 3.3 Relationship to neuropsychologist diagnosis

For the subset of patients who obtained a formal neuropsychological assessment, we repeated ANOVA and ROC analyses comparing test scores to the neuropsychologist’s diagnosis. All tests were significantly associated with diagnosis (*p* < 0.001, a one-way ANOVA; Supplementary Figure S2). For identifying cognitive impairment, the ROC AUC for SATURN (0.95) was numerically higher than that of most other tests (Supplementary Table S3). For identifying those with dementia, SATURN’s ROC AUC (0.84) was comparable to that of other tests (range 0.78–0.89) (Supplementary Table S3).

### 3.4 Correlations between SATURN and legacy tests

**Figure 3:**
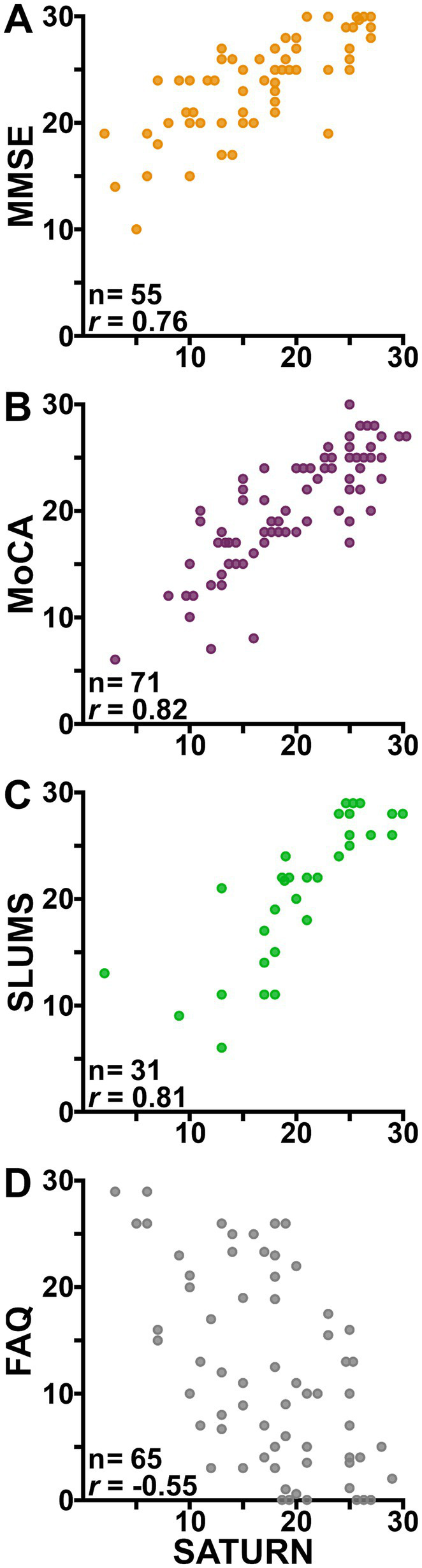
Self-Administered Tasks Uncovering the Risk of Neurodegeneration (SATURN) is well-correlated with legacy assessments. Scatterplots show the significant (all *p* < 0.001) associations between scores on SATURN (*x*-axes) and the Mini-Mental State Exam [MMSE, **(A)**], the Montreal Cognitive Assessment [MoCA, **(B)**], the Saint Louis University Mental Status [SLUMS, **(C)**] exam, and the Functional Activities Questionnaire [FAQ, **(D)**]. Where two or more patients had the same pair of test scores, data points are jittered by one-third of a test point to prevent overlap.

SATURN scores were well-correlated with those from the MMSE, MoCA, SLUMS, and FAQ (Figure 3; each *p* < 0.001). Those relationships were all linear (*p* > 0.08 for the quadratic term). Neither patient sex (*p* > 0.07) nor level of education (*p* > 0.10) improved the prediction of those legacy measures based on SATURN scores. Age improved prediction of the MoCA based on SATURN scores (*p* = 0.01; partial *R*^2^ = 0.09), such that if one’s SATURN score was unchanged over a 10-year period, MoCA would be expected to worsen by 1.2 ± 0.5 points in that same decade. For the other legacy tests (MMSE, SLUMS, and FAQ), age did not improve prediction based on SATURN scores (*p* > 0.10). Only limited testing was possible for the influence of ethnicity on the relationships between SATURN and the legacy tests: A high proportion of patients were identified as non-Hispanic White (84% of those for the SATURN vs. MMSE comparison, 80% vs. MoCA, 93% vs. SLUMS, and 81% for SATURN vs. FAQ). After binarizing demographic information—grouping patients based on whether they identified as non-Hispanic White—we found no evidence on the correlations between SATURN and any other test (*p* > 0.20).

Where multiple legacy test values were available for the same patient, the pair of values closest in time was used to calculate correlations between the legacy tests and between the first and second uses of the MMSE, MoCA, and SLUMS. Those three tests, along with SATURN, have middling power to predict FAQ (Supplementary Table S5). Otherwise, there is no obvious space for SATURN to improve: Its correlations with MoCA (*r* = 0.82) and SLUMS (*r* = 0.81) numerically exceed the test–retest correlations of those tools (*r* = 0.76 and *r* = 0.57, respectively).

### 3.5 Score prediction

When our approach was applied to preexisting repository data comparing SATURN to the MoCA (Bissig et al., 2020a), the resulting 50% prediction interval successfully captured 49% of new cases. The reverse—“predicting” pre-existing data with the new—was also successful, with 71% of cases falling within the prediction interval. For the MoCA, we combined the past (Bissig et al., 2020a) and present data to generate the predictions in Table 2.

**Table 2:** Prediction of MoCA, MMSE, and SLUMS scores using SATURN.

SATURN	MoCA		MMSE		SLUMS	
	est	50%P	est	50%P	est	50%P
0	*5*	*0–9*	*17*	*14–19*	*5*	*0–10*
1	*5*	*0–9*	*17*	*14–19*	*5*	*0–10*
2	*6*	*0–9*	*17*	*15–19*	*6*	*0–10*
3	*6*	*2–10*	*17*	*15–20*	*6*	*0–11*
4	*7*	*3–10*	*18*	*16–20*	*7*	*0–11*
5	*8*	*4–11*	*18*	*16–20*	*7*	*0–12*
6	*9*	*5–12*	*19*	*16–21*	*8*	*2–12*
7	*9*	*6–12*	19	17–21	*9*	*3–13*
8	*10*	*7–13*	19	17–22	*10*	*5–13*
9	*11*	*8–14*	20	18–22	*11*	*6–14*
10	12	9–14	20	18–22	*12*	*7–15*
11	13	10–15	21	19–23	*12*	*9–16*
12	13	11–16	21	19–23	*13*	*10–16*
13	14	12–17	22	20–24	*14*	*11–17*
14	15	13–17	22	20–24	*15*	*12–18*
15	16	13–18	23	21–25	*16*	*13–19*
16	17	14–19	23	21–25	*17*	*14–20*
17	18	15–20	24	22–26	18	15–20
18	19	16–21	24	22–26	19	16–21
19	19	17–21	25	23–27	20	17–22
20	20	18–22	25	23–27	21	18–23
21	21	19–23	26	24–28	22	19–24
22	22	20–24	27	25–28	23	20–25
23	23	21–25	27	25–29	23	21–26
24	24	22–26	28	26–30	24	22–26
25	25	23–27	28	26–30	25	23–27
26	26	24–27	29	27–30	26	24–28
27	27	25–28	29	27–30	27	25–29
28	27	26–29	30	28–30	28	26–30
29	28	27–30	30	28–30	29	27–30
30	29	27–30	30	29–30	30	28–30

Assuming that the legacy test scores are roughly normally distributed at each SATURN score, the mean estimate (est) would approximate the median, and the 50% prediction interval (“50%P”) would approximate the upper and lower quartiles. Therefore, if a specialty clinic required a MoCA ≥ 17 before prescribing an anti-amyloid therapy such as lecanemab, that clinic could use a referring provider’s SATURN score to predict that a patient likely (~75% chance) would have an eligible MoCA with a SATURN of 19 but likely would not have (~25% chance) with a SATURN of 13 or 14. Those assumptions, as well as the estimates themselves, are most tenuous when ≤ 5 people with that legacy score have had a scorable (Bissig et al., 2020b) SATURN result at least as low as the tabled value (italics), especially if no people have had a result that low (gray).

## 4 Discussion

In this retrospective study of SATURN’s real-world clinical use, it was an excellent predictor of clinical diagnosis. We found strong correlations between SATURN and legacy cognitive assessment tools: the MMSE, MoCA, SLUMS, and FAQ. These findings extend prior prospective validation work (Bissig et al., 2020b) by demonstrating that SATURN performs comparably to established cognitive screening instruments in routine clinical practice. Taken together, these results argue that SATURN is a scalable and clinically valuable cognitive screening tool.

Our findings shed light on the more extensive potential of computerized cognitive assessments in routine clinical practice. The patients observed in our subspecialty clinic were usually tested multiple times (Figure 1). We typically used SATURN once, in place of one legacy assessment, with great success. However, this may not generalize to a care pathway that tries to omit paper-and-pencil testing altogether. Digital platforms can create barriers to engaging elderly patients. Recent literature has highlighted the importance of patient perceptions in the adoption of emerging technologies for cognitive and functional assessments. For instance, older adults with limited knowledge of technology, including AI, tended to hold negative, distrustful attitudes toward its use (Giannouli, 2023a). This outlook was influenced by low familiarity with and perceived autonomy over testing—barriers to integrating technology into clinical practice (Giannouli, 2023a). While the current implementation of SATURN does not use AI, such findings are relevant to the future development of digital cognitive assessment platforms such as SATURN. Much like the electronic version of the Self-Administered Gerocognitive Examination (eSAGE) (Kawakami et al., 2024), behavioral features such as time spent on an instruction screen can be extracted from SATURN. With larger sample sizes, machine learning may help refine that additional information into a more accurate test, but this is only valuable if test engagement *and acceptance* are preserved. For the clinician counseling a patient and family on test results, mistakes on a paper-and-pencil test may be more tangible and intuitive than, for instance, latency and graphomotor metrics from the digital clock drawing test (Dion et al., 2020). Consideration of these factors is important when deploying scalable, technology-based cognitive assessments, particularly across diverse populations with varying levels of digital literacy and trust in automated systems. Reassuringly, the current implementation of SATURN is generally reported to be easy to use and often preferred over the MoCA (Bissig et al., 2020b).

Whatever caution one has for digital assessments, there is a clear need to improve upon paper-and-pencil tests. In addition to our initial motives for this project (e.g., addressing underutilization), digital assessments offer new ways to improve the detection of mild cognitive impairment. One incremental approach adds behavioral metrics to tools initially developed as paper-and-pencil tests (e.g., Dion et al., 2020; Kawakami et al., 2024). Moving beyond digital adaptations to legacy tests may be fruitful: for instance, spatial navigation deficits are early indicators of cognitive decline and dementia but are not easy to probe with paper-and-pencil tests. There is no single standardized spatial orientation test, and many older tools have limitations on ecological validity and scalability. New virtual tools appear useful in detecting declines in spatial orientation (Tragantzopoulou and Giannouli, 2024). Since SATURN is modeled after legacy paper-and-pencil tests, its integration into a clinical workflow may be a stepping stone toward the clinical use of less familiar but high-value digital assessment tools.

If considered in isolation, this study has two significant weaknesses. Foremost, the screening tests used for each patient depended on the clinician’s discretion. The consistency of the present findings with prior studies (Bissig et al., 2020b; Tagliabue et al., 2023) is reassuring. For instance, in the prospective validation wherein all were tested using SATURN, ROC curves suggested an optimal cutoff score for identifying impairment that was the same as in the present study (<24), with comparable sensitivities (82% vs. 86%) and specificities (92% vs. 96%) (Bissig et al., 2020b and Supplementary Table S2). The other significant weakness is that the neurologist (D.B.) was aware of at least some test results at the time of diagnosis. Reassuringly, re-running analyses based on the diagnosis of an independent neuropsychologist did not substantively change findings (Supplementary Table S3; Supplementary Figure S2).

Our focus was on the performance characteristics of SATURN and the legacy paper-and-pencil tests in routine clinical practice. When used as a screening tool, the etiology of cognitive impairment may be unknown, as was true for at least one-third of our heterogeneous sample (Table 1). Since a majority of participants did not undergo biomarker confirmation and only a minority had a comprehensive neuropsychological evaluation (Supplementary Table S3), we did not conduct subgroup analyses of cognitive test performance according to etiology. Legacy test scores understate the degree of impairment in some etiologies (e.g., behavioral variant of frontotemporal dementia vs. Alzheimer’s disease; Deutsch et al., 2016; Ranasinghe et al., 2016). SATURN is modeled after those legacy tests and may therefore share that weakness.

SATURN is a free, self-administered, public-domain cognitive assessment developed to minimize barriers to cognitive screening. The majority of those diagnosed with dementia are never tested with one of the well-regarded paper-and-pencil assessments (Nguyen et al., 2022) that SATURN reasonably predicts—the MMSE, MoCA, or SLUMS. The present findings argue that SATURN can add similar clinical value in situations where a legacy test is postponed or rejected. However, SATURN’s value will ultimately depend on the way a health system uses such tests. In one real-world care model (Weber et al., 2025), a specialty clinic assessing patients for lecanemab requires an MMSE, MoCA, or SLUMS score from the referring provider to facilitate triage. The specialist repeats at least one of those tests as part of a comprehensive assessment, which is prudent: The initial test results might have been influenced by evolving general medical conditions (broadly including polypharmacy and sleep disturbance), and the neurodegenerative process may have worsened since the referral. In that care model, SATURN might be of use to the referring provider, substituting for one of the legacy tests. We found that SATURN is no worse than the MoCA at predicting a MoCA score on a different date (Supplementary Table S5). By aggregating prior prospective data (Bissig et al., 2020b) with the current retrospective data, we have tabulated actionable, narrow predictions of MoCA scores based on SATURN (Table 2). Indeed, since completing the present analyses, we have begun asking for a SATURN sc2ore when no other comparable score (MMSE, MoCA, or SLUMS) is provided in a referral. A health system may value deploying SATURN more broadly, perhaps remotely (Tagliabue et al., 2023), to increase the frequency of cognitive screening and identify those who merit face-to-face reassessment using a legacy test. SATURN may thereby supplement those tests in the diagnosis of neurocognitive disorders, as we demonstrate in this study a strong association among SATURN score, neurologist diagnosis, and neuropsychologist diagnosis. However, we do not envision SATURN as a *replacement* for clinician- or neuropsychologist-administered cognitive assessments, particularly in specialized memory clinic settings. It is best to observe the patient engagement with a test and witness the types and magnitudes of his or her errors during the test before integrating those results into a clinical impression. Moreover, legacy tests benefit from decades of normative data accumulated across multiple demographics, including age, language, education, and other factors that influence test performance.

How would a clinician start using SATURN? To date, there is no “app” version of SATURN, although the German-language digiDEM-SCREEN (Zeiler et al., 2025b) may be available soon. Public domain implementations of SATURN exist for Python 2.5 (Bissig et al., 2020a) and PsychoPy. While PsychoPy can be translated into JavaScript and hosted online (Giaquinto et al., 2024), we commissioned another online version of SATURN,^1^ currently hosted at http://www.saturn-test.com.

## Data Availability

Publicly available datasets were analyzed in this study. This data can be found at: DOI: 10.5061/dryad.02v6wwpzr; DOI: 10.5061/dryad.j9kd51cq4.

## Footnotes

1
http://www.github.com/Operation-Cassini/
