# Supplementary material for: Retrospective validation of SATURN, a public domain self-administered cognitive screening test
Source: Front Psychol. 2026 May 18;17:1801901. doi: 10.3389/fpsyg.2026.1801901 (PMC13222788; doi:10.3389/fpsyg.2026.1801901)

<u>TABLE S1:</u> Demographic and diagnostic data automatically extracted from the electronic medical record indicates that the subset of charts selected for manual review was representative of the whole. For those *not* tested with SATURN (see left half of Figure 1), we wanted to gauge whether the charts we manually reviewed were representative of the whole. Tabled values are counts (#), percentages (analyzed with Chi-squared; Cohen's W), and means (with (standard deviations) analyzed with t-test; Cohen's d). Manual chart review often revealed missing data. For instance, level of education was automatically extracted from only 39% of those 470 patients who eventually received a manual chart review. Manual chart review uncovered level of education in 89% of those 470 patients. For the subset included in the primary analyses (main text Table 1) level of education was documented in 96%. Only for this comparison of automatically extracted data do we rely on International Classification of Diseases, 10th Revision (ICD-10) codes for the index event. * Combines chart entries of "Other", and entries with prevalence < 2%.

		Manually Reviewed?		*p*	effect size
		No	Yes		
Patients	#	414	470		
Age	yr	74 (11)	73 (12)	0.3	0.08
Women	%	55	59	0.2	0.04
Race Documented	%	84	85	0.7	0.01
African American, Asian, White, Other *	%	5, 5, 77, 13	6, 7, 75, 12	0.5	0.05
Ethnicity Documented	%	82	81	0.8	0.01
Hispanic/Latino	%	8	8	1.0	0.00
Education Documented	%	36	39	0.5	0.02
Education	yr	15 (4)	15 (3)	1.0	0.00
ICD10 Dementia Diagnosis	%	42	41	0.9	0.01
ICD10 Alzheimer's Diagnosis	%	20	15	0.053	0.06

TABLE S2: Receiver operating characteristic and precision-recall analyses of test performance compared to neurologist diagnosis. All tests have a possible range of 0 to 30. Unlike the others, higher Functional Activities Questionnaire (FAQ) scores imply greater impairment. For this table, we therefore subtract FAQ score from 30. Tabled values reflect beeswarm plots in Figure S1. In the top half of the table, scores greater than or equal to the cutoff favor a diagnosis of "normal". *Abbreviations: Area Under the Curve (AUC), Self-Administered Tasks Uncovering the Risk of Neurodegeneration (SATURN), Montreal Cognitive Assessment (MoCA), Mini Mental State Exam (MMSE), Saint Louis University Mental Status (SLUMS) exam, and Functional Activities Questionnaire (FAQ), Mild Cognitive Impairment (MCI).* * indicates F_1 is highest at the "baseline" of any FAQ score; F_1, precision, and recall were instead calculated for FAQ > 0.

test	#without/with diagnosis	Receiver Operator Characteristic Curve				Precision-Recall Curve				
		AUC	best cutoff < favors Dx	sens. (%)	spec. (%)	AUC	best cutoff < favors Dx	F_1	precision (%)	recall (%)
...for identifying IMPAIRED (MCI or dementia) vs NOT										
SATURN	23 /94	0.95	24	86	96	0.99	26	0.94	93	96
MoCA	47 /179	0.93	24	85	85	0.98	27	0.94	89	99
MMSE	18 /191	0.91	28	84	89	0.99	30	0.96	95	96
SLUMS	27 /92	0.93	24	84	85	0.98	28	0.93	88	99
30 - FAQ	18 /168	0.79	25	79	61	0.97	*30	0.94	91	96
...for identifying DEMENTIA vs NOT (cognitively normal or MCI)										
SATURN	46 /71	0.90	19	93	76	0.87	19	0.81	72	93
MoCA	132 /94	0.87	20	73	80	0.83	22	0.74	65	87
MMSE	92 /117	0.87	25	80	77	0.91	25	0.81	82	80
SLUMS	75 /44	0.91	20	86	80	0.87	21	0.78	69	91
30 - FAQ	94 /92	0.86	18	72	82	0.85	23	0.79	69	95

<u>TABLE S3:</u> Receiver operating characteristic and precision-recall analyses of test performance compared to *neuropsychologist* diagnosis. For the subset of patients who had a formal neuropsychological assessment within ±365 days of the index event, Table S2 analyses are repeated using neuropsychologist diagnosis instead of neurologist diagnosis. Here, if a legacy test was used more than once for a patient, we analyzed the score obtained closest in time to the neuropsychological assessment. Tabled values reflect beeswarm plots in Figure S2. Abbreviations are identical to Table S2. * indicates F_1 is numerically highest at the "baseline" of any MMSE score or any FAQ score; F_1, precision, and recall were instead calculated for MMSE < 30 and FAQ > 0. † this cutoff is a non-integer due to a patient with dementia who had one point omitted on the SLUMS (21/29, transformed to 21.72/30 for planned analyses). Recalculating the precision-recall curve either granting that point (22/30) *versus* not (21/30) has negligible impact on AUC and F_1 (change by < 0.01), but shifts the other metrics (respectively, cutoff = 21, precision = 94%, recall = 80% *versus* cutoff = 22, precision = 85%, recall = 87%).

test	#without/with diagnosis	Receiver Operator Characteristic Curve				Precision-Recall Curve				
		AUC	best cutoff < favors Dx	sens. (%)	spec. (%)	AUC	best cutoff < favors Dx	F_1	precision (%)	recall (%)
...for identifying IMPAIRED (MCI or dementia) vs NOT										
SATURN	6 /32	0.95	24	81	100	0.99	28	0.94	89	100
MoCA	11 /63	0.90	24	79	82	0.98	27	0.96	93	100
MMSE	3 /57	0.89	29	84	100	0.89	*30	0.93	97	93
SLUMS	5 /29	0.96	25	86	100	0.99	29	0.95	91	100
30 - FAQ	3 /57	0.71	26	77	67	0.98	*30	0.95	95	95
...for identifying DEMENTIA vs NOT (cognitively normal or MCI)										
SATURN	18 /20	0.84	21	80	78	0.86	21	0.80	80	80
MoCA	41 /33	0.78	22	79	63	0.76	24	0.71	58	91
MMSE	23 /37	0.80	26	70	78	0.86	29	0.80	71	92
SLUMS	14 /20	0.89	22	85	86	0.94	†21.8	0.87	89	85
30 - FAQ	25 /35	0.84	23	89	68	0.88	23	0.83	79	89

TABLE S4: Direct comparison of the receiver operating characteristics of test score versus neurologist diagnosis. In contrast to Table S2, each row of this table limits analysis to patients who have values for both tests. For instance, of the 23 cognitively normal patients and 94 cognitively impaired patients who were tested with SATURN (Table S2) only 16 and 55, respectively, also were tested with the MoCA within ±365 days of the index event. The *p*-value compares the area under the curve (AUC) for the two tests named in each row, as calculated with the roc.test function in the R (version 4.4.1) library pROC. Abbreviations are the same as in Table S2.

tests	#without/with diagnosis	SATURN's AUC	other AUC	*p*
...for identifying IMPAIRED (MCI or dementia) vs NOT				
SATURN & MoCA	16 /55	0.95	0.90	0.12
SATURN & MMSE	4 /57	0.89	0.93	> 0.4
SATURN & SLUMS	9 /22	0.95	0.96	> 0.8
SATURN & FAQ	7 /61	0.94	0.78	0.0045
...for identifying DEMENTIA vs NOT (cognitively normal or MCI)				
SATURN & MoCA	45 /26	0.91	0.87	0.19
SATURN & MMSE	29 /32	0.91	0.84	0.10
SATURN & SLUMS	23 /8	0.90	0.97	0.16
SATURN & FAQ	39 /29	0.91	0.91	> 0.8

<u>TABLE S5:</u> Test score correlations. We quantify the correlation coefficients (r) and number of cases (n) shared between legacy tests, SATURN, and the FAQ, as well as the correlations between the first and second use of each legacy test (*grey*). Aside from the test-retest correlation for SLUMS (p = 0.018), every other correlation was significant after appropriate thresholding for multiple comparisons (p < 0.001). In parenthesis, we show the inter-quartile interval of the absolute value of days (d) elapsed between one test and the other. Test abbreviations are the same as in Table S2.

	SATURN	MoCA	MMSE	SLUMS
FAQ	r -0.55<br>n 65<br>(0 - 181 d)	r -0.60<br>n 120<br>(0 - 93 d)	r -0.50<br>n 123<br>(0 - 112 d)	r -0.45<br>n 58<br>(0 - 37 d)
SATURN		r 0.82<br>n 71<br>(95 - 182 d)	r 0.76<br>n 58<br>(28 - 182 d)	r 0.81<br>n 31<br>(100 - 183 d)
MoCA		*r 0.76*<br>*n 71*<br>*(96 - 265 d)*	r 0.77<br>n 123<br>(46 - 172 d)	r 0.82<br>n 62<br>(59 - 182 d)
MMSE			*r 0.87*<br>*n 77*<br>*(105 - 253 d)*	r 0.74<br>n 50<br>(88 - 186 d)
SLUMS				*r 0.57*<br>*n 17*<br>*(168 - 199 d)*

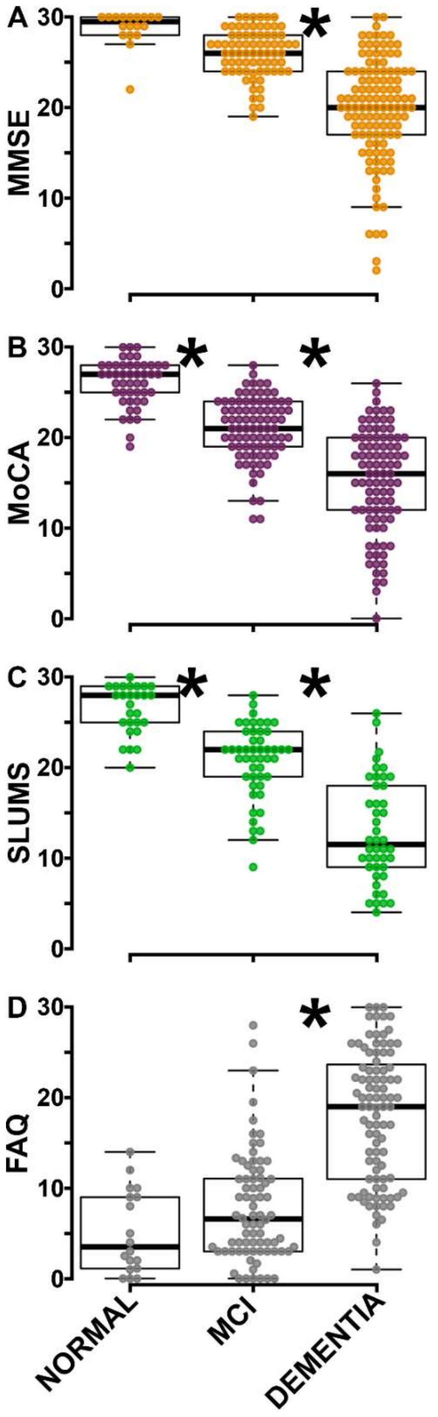

FIGURE S1: **Relationship between *neurologist* diagnosis and test score.** Sample sizes here match Table S2. For instance, the orange points in panel A reflect the availability of Mini Mental State Exam (MMSE) scores from n = 18 normal patients, n = 117 patients with dementia, and (subtracting 117 with dementia from 191 with impairment yields) n = 74 patients with mild cognitive impairment (MCI). All tests were significantly associated with diagnosis ($p < 0.001$ for each test's one-way ANOVA) and effect sizes were numerically higher for the Montreal Cognitive Assessment (MoCA, panel B, $\eta^2 = 0.48$), Saint Louis University Mental Status (SLUMS, panel C, $\eta^2 = 0.58$), and Functional Activities Questionnaire (FAQ, panel D, $\eta^2 = 0.40$), than for the MMSE ($\eta^2 = 0.36$). The group differences driving these effects were explored with Tukey's Honestly Significant Differences (HSD) test. For each test, normal and dementia groups clearly differed (all $p < 0.001$; for MMSE, MoCA, SLUMS and FAQ, respectively, Cohen's d = 1.67, 2.23, 2.81, and 1.86). Normal and MCI groups had similar scores on the MMSE (p = 0.057; d = 1.10) and FAQ (p = 0.28; d = 0.46), but had significantly different scores (*; each $p < 0.001$) on the MoCA (d = 1.59) and SLUMS (d = 1.54). For each test, MCI and dementia groups were significantly different (all $p < 0.001$; for MMSE, MoCA, SLUMS and FAQ, respectively, Cohen's d = 1.31, 1.22, 1.59, and 1.51). Data from Self-Administered Tasks Uncovering the Risk of Neurodegeneration (SATURN) are shown in the main text (Figure 2).

<u>FIGURE S2</u>: Relationship between *neuropsychologist* diagnosis and test score. Here, if a legacy test was used more than once for a patient, we analyzed the score obtained closest in time to the neuropsychological assessment. Abbreviations and panels are identical to those in Figure S2, now with data from SATURN (panel E), which includes two "unscorable" patients (Xs). An "unscorable" SATURN test supports the diagnosis of dementia (Bissig et al., 2020b). Sample sizes here match Table S3. For instance, the orange points in panel A reflect the availability of MMSE scores from n = 3 normal patients, n = 37 patients with dementia, and (subtracting 37 with dementia from 57 with impairment yields) n = 20 patients with MCI. All tests were significantly associated with diagnosis (p < 0.001 for each test's one-way ANOVA). Effect sizes were numerically higher for MoCA (panel B, η^2 = 0.34), SLUMS (panel C, η^2 = 0.44), FAQ (panel D, η^2 = 0.33), and SATURN (η^2 = 0.42) than for the MMSE (η^2 = 0.24). The group differences driving these effects were explored with Tukey's HSD test. Normal and dementia groups had similar FAQ scores (p = 0.067; Cohen's d = 1.23) but differed for the MMSE (p = 0.021; d = 1.50) as well as the MoCA, SLUMS, and SATURN (each p < 0.001; respectively, d = 1.84, 1.94, and 2.03). Normal and MCI groups had similar scores on the MMSE (p = 0.57; d = 0.86)**,** SLUMS (p = 0.32; d = 1.92)**,** FAQ (p = 1.0; d = 0.02), and SATURN (p = 0.098; d = 1.54), but differed (*) on the MoCA (p = 0.0070; d = 1.37). MCI and dementia groups differed (*) on all tests (for MMSE, MoCA, SLUMS, FAQ, and SATURN, respectively, p = 0.0014, 0.0019, 0.0053, < 0.001, and 0.012, while d = 1.01, 0.86, 1.28, 1.39, and 1.04)

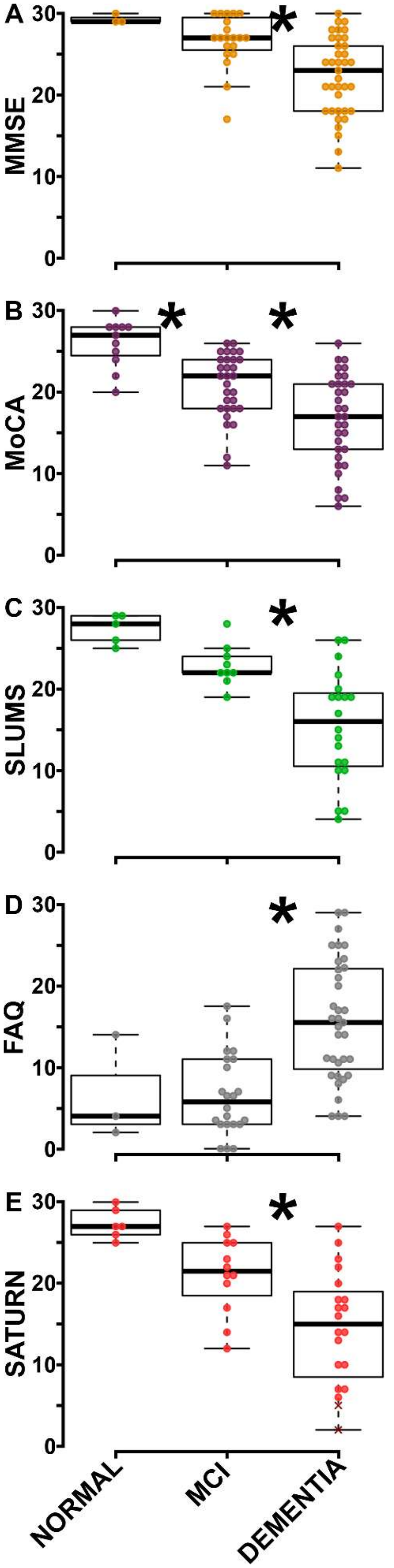

Supplement: Supplementary file 1 [file Data_sheet_1.pdf]
